# Polyethylene Glycol-Carbon Nanotubes/Expanded Vermiculite Form-Stable Composite Phase Change Materials: Simultaneously Enhanced Latent Heat and Heat Transfer

**DOI:** 10.3390/polym10080889

**Published:** 2018-08-09

**Authors:** Yong Deng, Mingyue He, Jinhong Li, Zhiwei Yang

**Affiliations:** 1 Beijing Key Laboratory of Materials Utilization of Nonmetallic Minerals and Solid Wastes, National Laboratory of Mineral Materials, School of Materials Science and Technology, China University of Geosciences, Beijing 100083, China; dengyong@cugb.edu.cn (Y.D.); yangzhiwei@cugb.edu.cn (Z.Y.); 2 School of Gemology, China University of Geosciences, Beijing 100083, China

**Keywords:** composite phase change materials, enhanced latent heat, enhanced thermal conductivity, surface interaction

## Abstract

Polyethylene glycol (PEG)-carbon nanotubes (CNTs) with expanded vermiculite (EVM) form-stable composite phase change materials (PCE-CPCMs) were constructed via the efficient synergistic effect between EVM and CNTs. The resultant material demonstrated simultaneously enhanced latent heat and heat transfer. The unique EVM pore structure and CNTs surfaces contributed to the form stability of PCE-CPCMs. The adsorption capacity was 77.75–81.54 wt %. The latent heat of the PCE-CPCMs increased with increasing CNTs content due to the decreasing inhibition effect of EVM and the increasing adsorption capacity of PEG, which was 83.9 J/g during melting and 104.2 J/g during solidification for PCE7.09. The pore confinement and surface EVM interactions inhibited the heat storage capacity of the PCE-CPCMs. Moreover, the inhibition effect on the heat storage capacity of PCE-CPCMs during the melting process was stronger than during solidification due to the crystallization-promoting effect. The heat transfer of PCE-CPCMs was significantly enhanced by the CNTs filler (0.5148 W/(m·K) for PCE7.09) due to the decrease in interfacial thermal resistance and the formation of rapid thermally conductive pathways. Fourier transform infrared spectroscopy, thermogravimetric analysis, and thermal cycles test results confirmed that the PCE-CPCMs exhibited excellent chemical compatibility, thermal stability, and reliability.

## 1. Introduction

Phase change materials (PCMs) are interesting materials because they can absorb and release a large amount of latent heat from the environment, by changing phase or structure, and provide large heat storage density, thus increasing the efficiency and sustainability of available thermal resources [1]. The organic and eutectic PCMs, such as polyethylene glycol (PEG), paraffin, and fatty acid, have been extensively studied due to their suitable and adjustable phase change temperature, large latent heat, excellent chemical stability, negligible supercooling behavior, and lack of corrosiveness and phase separation. However, the low thermal conductivity of organic PCMs results in low energy conversion efficiency, and the poor form stability leads to a leakage issue after solid–liquid transitions. These drawbacks severely restrict the practical applications of organic PCMs, which could include for building-integrated solar energy conservation, building energy conservation, and thermal management for electronic devices [2,3].

Efforts have been directed at simultaneously preventing leakage and enhancing heat transfer rates of organic PCMs [4,5]. Numerous thermally conductive filler-dispersed form-stable composite materials PCMs (fs-CPCMs) have been developed, including PEG/AlN/SiO_2_ [6], PEG/graphite nanoplatelets/polymethyl methacrylate [7], PEG/carbon fiber/SiO_2_ [8], PEG/carbon nanotubes (CNTs)/SiO_2_ [9], PEG/silver nanoparticles/diatomite [10], and PEG/boron nitride/graphene oxide [11]. The thermally conductive fillers play a crucial role in improving and adjusting the thermophysical properties of fs-CPCMs, such as phase change temperature, latent heat, heat capacity, heat storage density, and thermal conductivity. The filler size, shape, concentration, and surface properties might affect the heat storage behavior and heat transfer efficiency of fs-CPCMs. For example, Zhang et al. [7] discovered that the thermal conductivity (0.253–2.339 W/(m·K)) of prepared PEG/graphite nanoplatelets/polymethyl methacrylate fs-CPCMs was enhanced by the graphite nanoplatelets (0–8 wt %). However, the latent heat of fs-CPCMs decreased with increasing graphite nanoplatelet content. The latent heat decreased by a maximum of 5.23% during the melting process and 7.18% during solidification compared with no fillers. Yang et al. [11] reported that the thermal conductivity of PEG/boron nitride/graphene oxide fs-CPCMs significantly improved by introducing boron nitride fillers and graphene oxide (fs-CPCM containing 4 wt % graphene oxide and 30 wt % boron nitride: 3.00 W/(m·K), relative enhancement ratio compared with pure PEG: 900%), whereas the phase change enthalpies decreased with increasing boron nitride fillers. The phase change enthalpies decreased by 31.11% during the melting process and 31.29% during solidification compared with 4 wt % graphene oxide fs-CPCM). The decline in heat storage capacity mainly occurs due to the addition of thermally conductive fillers reducing the PEG content, and the fillers do not undergo a phase change within the test temperature range. Hence, balancing high thermal conductivity and the available latent heat of fs-CPCMs is necessary when being used in practical applications. Simultaneously maintaining large latent heat and high heat transfer rate for fs-CPCMs remains a challenge.

However, the CNTs showed the opposite result with graphite nanoplatelets and boron nitride fillers. Tang et al. [9] found the prepared PEG/CNTs/SiO_2_ fs-CPCMs containing 0.5–3 wt % CNTs showed higher latent heat than PEG/SiO_2_ fs-CPCM without fillers (melting process > 30.16%, solidification process > 9.15%), and the thermal conductivity of these fs-CPCMs was also increased, by a maximum of 28.97% compared with no fillers. Chen et al. [12] found that the enthalpy of paraffin/CNTs sponge composite PCMs increased almost linearly with increasing paraffin wax loading. Above a critical loading of 91 wt %, the enthalpy (138.2 J/g) was even higher than that of pure paraffin wax (136.0 J/g). The above results indicate that the presence of CNTs fillers has a positive influence on the heat storage behavior and heat transfer efficiency. The specific surface area of single-walled CNTs is larger than that of multi-walled CNTs, which is beneficial for enhancing the adsorption capacity of PEG, whose maximum adsorption capacity can reach 98 wt % [13]. Moreover, Single-walled CNTs usually show higher axial thermal conductivity than multi-walled CNTs, so the former was selected as the thermally conductive fillers in this work.

Suitable fs-CPCMs encapsulation materials play a key role in preventing leakage of organic PCMs and providing high heat storage capacity. The porous clay minerals—BN porous scaffolds—and expanded graphite-based fs-CPCMs demonstrated the large adsorption capacity of PCMs and excellent thermophysical properties [5,14,15,16,17]. Expanded vermiculite (EVM), a hydrous phyllosilicate mineral, has attracted attention due to its unique pore structures, high porosity, and large specific surface area, which create a large encapsulation space and the high heat storage capacity of organic PCMs. Moreover, good chemical inertness and compatibility, desirable thermal stability, non-flammability, non-toxicity, lightweight, low cost, and extensive sources are the notable advantages for use as this promising encapsulation material.

In this work, PEG served as the PCM for thermal energy storage. The CNTs were introduced into PEG as ideal thermally conductive fillers. Expanded vermiculite (EVM) was used as a form stabilizer. PEG-CNTs/EVM fs-CPCMs (PCE-CPCMs) were constructed. We expected that simultaneously enhanced form stability, latent heat, and heat transfer of PCE-CPCMs could be realized via the efficient synergistic effect between EVM and CNTs. The effects of the synergy between EVM and CNTs on heat storage behavior and heat transfer enhancement of PCE-CPCMs have been studied in detail, and the reason for this effect has been clarified. Additionally, the microstructure, chemical compatibility, heat storage and release properties, and thermal stability and reliability of PCE-CPCMs also are analyzed.

Based on previous research [18], a latent heat of at least 50 J/g was needed at an affordable cost for heat storage applications of composite PCMs, such as using a building-integrated solar thermal system. Yao et al. [19] reported expanded perlite-based composite PCMs wallboard (melting: 27.60 °C and 67.13 J/g; solidification: 23.56 °C and 67.06 J/g), which effectively reduced the indoor temperature. Fu et al. [20] prepared expanded perlite-based composite PCMs boards (87.44 J/g; 0.178 W/(m·K)), exhibiting good performance in increasing the thermal inertia and reducing the indoor temperature fluctuation. Jeong et al. [21] prepared gypsum board with 30 wt % fs-CPCMs (melting: 20–65 °C and 15.240–6.793 J/g; solidification: 14–62 °C and 13.08–4.506 J/g; 0.60 W/(m·K)), which could be used as a heat storage building material to establish heat storage structures for enhancing thermal efficiency. The properties of PCE-CPCMs are expected to be comparable to the above composite PCMs, thereby providing a valuable reference for future practical heat storage applications as a potential candidate in building-integrated solar energy conversion systems. The main applications are as follows: (1) PCE-CPCMs and gypsum slurry are mixed to prepare gypsum board, (2) PCE-CPCMs serve as aggregate (sand replacement) for developing structural-functional integrated cement or concrete, and (3) PCE-CPCMs are directly pressed into the boards as heat storage units.

## 2. Experimental Section

### 2.1. Materials

PEG (C.P.) with an average relative molecular weight of 2000 was purchased from Xilong Chemical Reagent Co., Ltd., Guangzhou City, Guangdong Province, China. EVM was obtained from Lingshou County, Shijiazhuang City, Hebei Province, China, and its chemical composition is shown in Table 1. Single-walled CNTs (purity: >90%; bundle diameter: ~40 nm; length: 5–30 μm) were provided by Suzhou Carbon-Rich Graphene Technology Co., Ltd., Suzhou City, Jiangsu Province, China.

### 2.2. Preparation of PCE-CPCMs

The PCE-CPCMs were prepared via the physical blending and impregnation method reported in our previous work (stirring process: 70 °C for 2 h, impregnation: 70 °C for 4 h, then drying oven: 70 °C) [22]. The components of the obtained PCE-CPCMs are shown in Table 2. PCE5.20C was obtained after the PCE5.20 was heated and cooled 100 times between 0 and 70 °C at a rate of 20 °C/min. Compared with the adsorption capacity of PCE-CPCMs without CNTs (68.59 wt %) [23], the PCE1.59, PCE3.30, PCE5.20, and PCE7.09 reached 77.75, 79.31, 81.53, and 81.54 wt %, respectively, and the corresponding weight fractions of CNTs were 1.59, 3.30, 5.20, and 7.09 wt %, respectively. Usually, the adsorption capacity of composite PCMs decreases with increasing thermal conductivity enhancement filler content. However, the PCE-CPCMs showed the opposite result due to the efficient synergistic effect between EVM and CNTs. Both EVM and CNTs had large specific surface areas, which were beneficial to the adsorption and form stability of PEG.

### 2.3. Characterization

The EVM pore size distribution was determined by mercury intrusion porosimetry (Quantachrome Instruments, PoreMaster 60, Waltham, MA, USA) and N_2_ adsorption analyzer (Beishide Instrument, 3H-2000PS1, Beijing, China). The EVM, CNT, and PCE5.20 morphology was observed by using a scanning electronic microscope (SEM, HITACHI, S-4800, Tokyo, Japan). All samples were coated with gold particles before observing the increase in their electroconductivity. The CNTs and PCE5.20 microstructures were further analyzed by transmission electron microscope (TEM, FEI, Tecnai G2 F20 S-TWIN, Waltham, MA, USA). The chemical compatibility of PCE-CPCMs and PCE5.20C was examined using Fourier transform infrared spectroscopy (FT-IR, SHIMADZU FTIR8400, Kyoto, Japan) at a testing wavelength of 400–4500 cm^−1^. The phase change parameters for PEG, PCE-CPCMs, and PCE5.20C were obtained with a differential scanning calorimeter (DSC, TA Instrument, Q100, New Castle, DE, USA) during the temperature transition of 0–70–0 °C, at a heating/cooling rate of 5 °C/min, under a N_2_ atmosphere. The PCE-CPCMs thermal conductivity was measured using the transient hotline method (XIATECH, TC3100, Xi’an, Shaanxi, China) at 26 °C. All samples were pressed into two planes with an approximate size of 4.5 × 3.0 × 0.2 cm, and then the probe was placed in between the two planes. Each PCE-CPCMs sample was measured three times and the mean value was taken as the thermal conductivity. The time-temperature curves of PEG and PCE-CPCMs during the heating and cooling processes were measured using the constant temperature water bath method (melting: 65 °C, solidifying: 20 °C, multi-channels temperature recorder: TOPRIE TP720 (Shenzhen Toprank Electronics Co. Ltd., Shenzhen, Guangdong, China), Thermocouple: T, Record interval: 1 s). The thermal stability of PCE-CPs was measured using thermo-gravimetric analysis (TGA, TA Instrument, Q5000, New Castle, DE, USA) under heating from 30 to 600 °C, at a rate of 10 °C/min, under a N_2_ atmosphere.

## 3. Results and Discussion

### 3.1. Form-Stability Analysis

**Figure 1:**
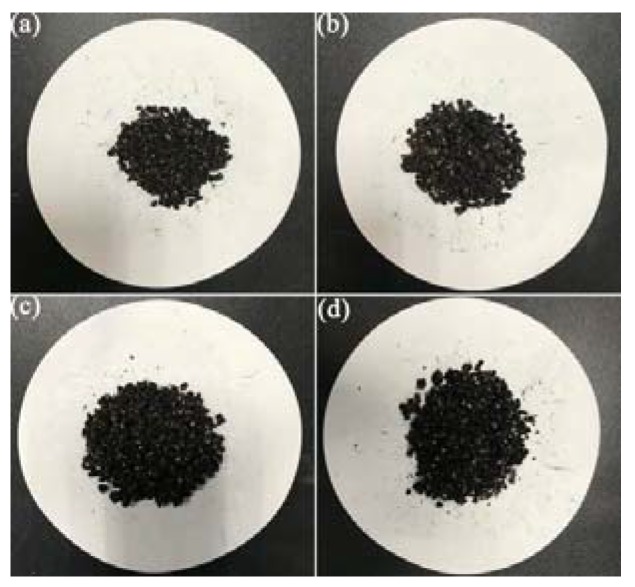
Photographs of polyethylene glycol (PEG)-carbon nanotubes (CNTs) with expanded vermiculite (EVM) form-stable composite phase change materials (PCE-CPCMs) with good form stability: (**a**) PCE1.59, (**b**) PCE3.30, (**c**) PCE5.20, and (**d**) PCE7.09.

**Figure 2:**
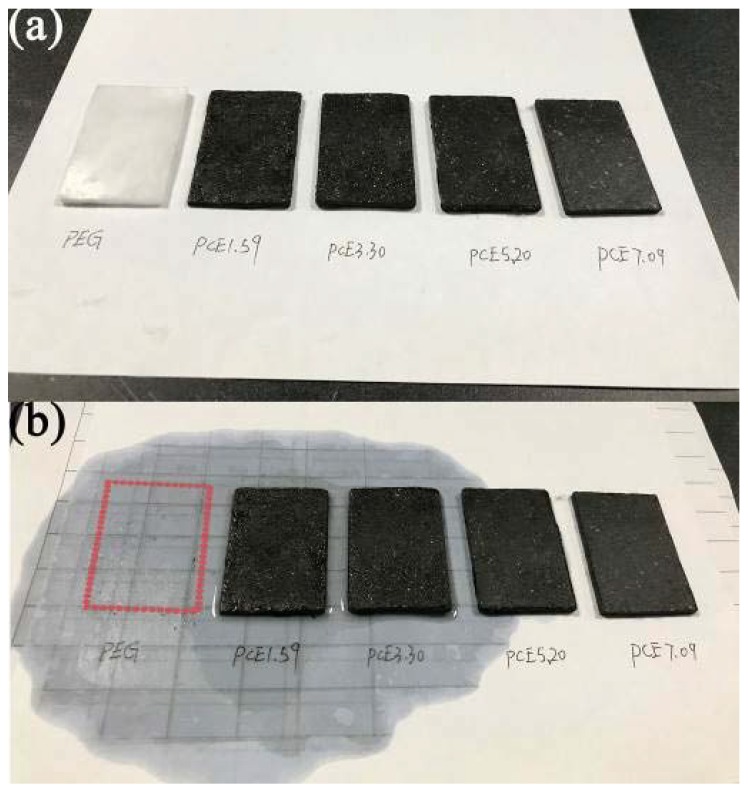
Form stability test of PEG and PCE-CPCMs board (approximately 4.5 × 3.0 × 0.2 cm): (**a**) before heat treatment and (**b**) after heating to 70 °C for 1 h.

The uniformly distributed PCE-CPCMs on the filter paper were placed into a drying oven and maintained at 70 °C for 1 h. After cooling to room temperature, the PCE-CPCMs were gathered together (Figure 1). No obvious leakage trace on the surface of filter paper was observed and the PCE-CPCMs maintained their shape in the solid state, indicating the PCE-CPCMs exhibited good form-stability, even though their adsorption capacity was higher than 77 wt %. To further test the form-stability of PCE-CPCMs, the samples were pressed into the boards (approximately 4.5 × 3.0 × 0.2 cm) (Figure 2a). After heating to 70 °C for 1 h, the PEG board completely leaked and an obvious trace was observed. However, the PCE-CPCMs still maintained their pre-heat treatment (Figure 2b), revealing excellent form-stability. The stable form of PCE-CPCMs was attributed to the efficient synergistic effect between EVM and CNTs. The unique EVM porous structure and large specific surface area of CNTs could strongly adsorb and restrict the melted PEG, thereby preventing the occurrence of leakage during the solid–liquid phase change.

### 3.2. Characterization of EVM Pore Structures

**Figure 3:**
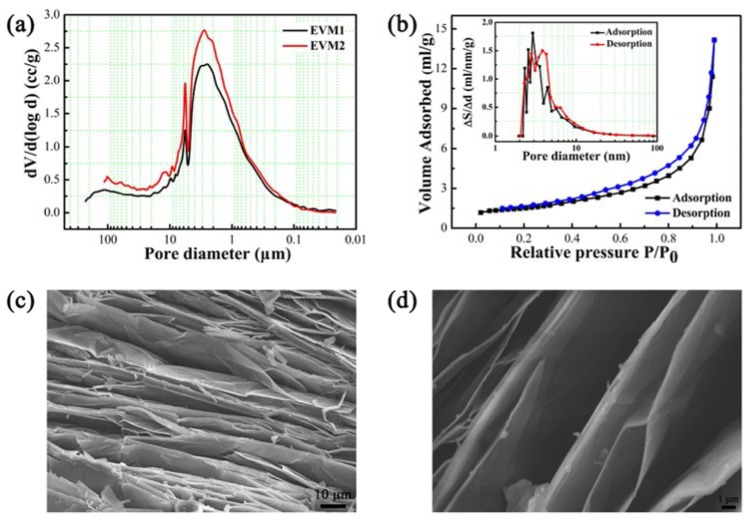
Characterization of EVM pore structures: (**a**) pore diameter distribution determined by mercury intrusion porosimetry (EVM1 and EVM2 represent the first and second measurement result, respectively); (**b**) nitrogen adsorption-desorption isotherms and the corresponding pore-size distribution curve (inset); and (**c**,**d**) SEM images of EVM (×1.0 k and ×5.0 k).

The EVM pore structures parameters played an important role in analyzing and predicating the heat storage capacity and behavior. The EVM pore size distribution curve, determined by mercury intrusion porosimetry, is shown in Figure 3a. The pore diameter was mainly distributed in the range of 30 to 50 μm and the most probable pore size was 2.8 μm, indicating that EVM could provide a large and multi-scale encapsulation space for PEG. To further analyze the EVM pore structure parameters, the nitrogen adsorption-desorption isotherms (type-IV) and pore-size distribution, calculated using the Barrett-Joyner-Halenda method, are shown in Figure 3b. The result indicated the presence of nanoscale pores, with the most probable pore size of 2.9 nm in adsorption and 3.8 nm in desorption, efficiently improved the thermo-physical properties of nanoconfined PCM [24,25]. Therefore, EVM was mainly composed of micron-scale as well as nanoscale pore structures, which could simultaneously provide large adsorption capacity and enhance the thermal performance of PCM, demonstrating that EVM is a promising encapsulation material for the preparation of efficient heat storage materials. Figure 3c,d show the SEM images of EVM. The EVM showed typical non-uniform multi-layered structures, which was consistent with the pore size distribution observed (Figure 3a,b). The unique pore structures were expected to contribute to the form stabilization of PCE-CPCMs.

### 3.3. Morphology and Microstructure of CNTs and PCE-CPCMs

**Figure 4:**
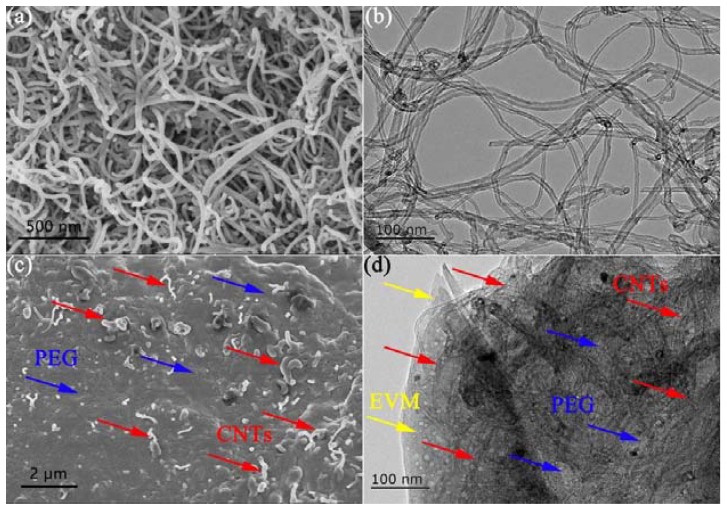
(**a**) SEM (×50.0 k) and (**b**) TEM images of CNTs; and (**c**) SEM (×10.0 k) and (**d**) TEM images of PCE5.20.

Figure 4 illustrates the SEM and transmission electron microscopy (TEM) images of CNTs and PCE5.20. The CNTs clearly had rough surfaces and were agglomerated and entangled, which could act as a thermal conductivity enhancement network for the highly efficient heat transfer of PCE-CPCMs. The length of the CNTs was approximately 5–30 μm, and the bundle diameter was ~40 nm (Figure 4a), which was further confirmed by the TEM result (Figure 4b). PEG-enwrapped CNTs were homogenously embedded in the pore structures and adsorbed on the surfaces of EVM, and the pore structures of EVM were almost completely occupied by the PEG-enwrapped CNTs (Figure 4c). The TEM image exhibited a similar result to the SEM analysis, as predicted (Figure 4d). Under the action of capillary force and surface tension, the synergistic effect between the EVM pore structures and the surfaces of CNTs could effectively prevent the leakage of the melted PEG, even though the adsorption capacity of PCE5.20 reached 81.53 wt %. Moreover, thermally conductive pathways formed by entangled CNTs network significantly contributed to the heat transfer enhancement of PCE-CPCMs.

### 3.4. Chemical Compatibility of PCE-CPCMs

**Figure 5:**
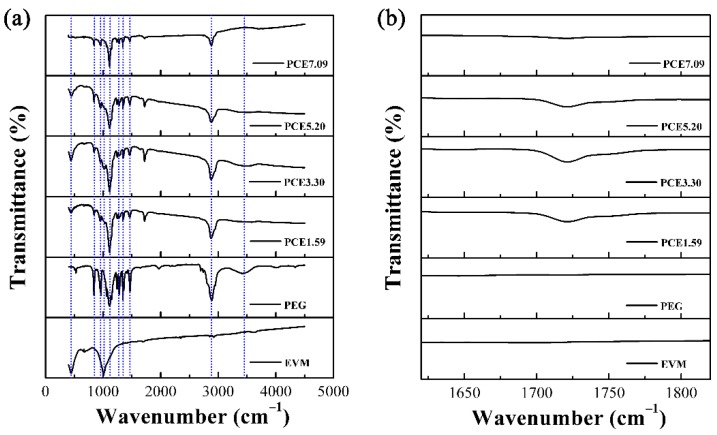
(**a**) Fourier transform infrared (FT-IR) spectra of EVM, PEG, and PCE-CPCMs, (**b**) enlarged at 1620–1820 cm^−1^.

The excellent chemical compatibility of composite PCMs contributed to maintaining the high latent heat after a large number of thermal cycles, or the chemical reaction between encapsulation material and PCM gradually caused composite PCMs to lose their heat storage capacity. The FT-IR spectra of EVM, PEG, and PCE-CPCMs are shown in Figure 5. As shown in the EVM spectrum, the peaks at 447 and 1011 cm^−1^ corresponded to the Si-O-Mg bending vibration, and Si-O-Si and Si-O-Al stretching vibrations, respectively [26]. In the PEG spectrum, the peaks at 842; 964 and 2889 cm^−1^; 1114 and 1242 cm^−1^; 1281, 1343, and 1467 cm^−1^; and 3421 cm^−1^ were attributed to the interim-CH_2_-group vibration, C-H stretching vibration of CH_2_, C-O stretching vibration, C-H bending vibration, and O-H stretching vibration, respectively [23]. All EVM and PEG absorption peaks were clearly observed in the PCE-CPCMs spectra and no new significant functional group appeared, except for the peak at 1720 cm^−1^ (C=O was formed due to the oxidation of PEG in the preparation of PCE-CPCMs) (Figure 5b). Moreover, the peaks of PCE-CPCMs showed a one-to-one correspondence with those of PEG and EVM. The above results confirmed the physical interaction among the components of PCE-CPCMs and demonstrated the desirable chemical compatibility of PCE-CPCMs.

### 3.5. PCE-CPCMs Heat Storage Behavior

**Figure 6:**
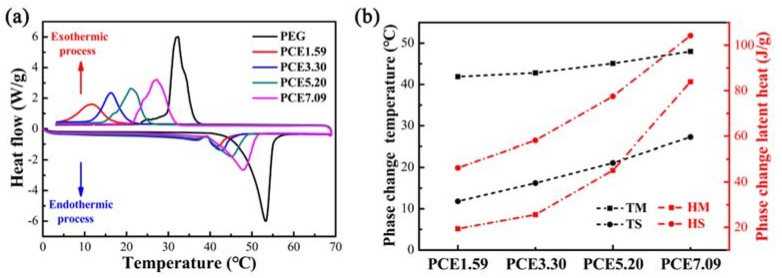
(**a**) Differential scanning calorimeter (DSC) curves of PEG and PCE-CPCMs and (**b**) the corresponding changes in phase change temperature and latent heat.

The effect of synergy between EVM and CNTs on the heat storage behavior of PCE-CPCMs was investigated by using DSC. The DSC curves of PEG and PCE-CPCMs are presented in Figure 6a, and the detailed phase change characteristic changes and values are shown in Figure 6b and Table 3, respectively. As shown in Figure 6a, the PCE-CPCMs exhibited similar endothermic and exothermic characteristics as PEG. However, the phase change temperature of all the PCE-CPCMs was lower than PEG, which could be explained by the Gibbs-Thomson equation and a physical interaction-induced decrease in phase change temperature [27,28,29,30,31]. Moreover, the DSC curves of both the melting and solidification processes of the PCE-CPCMs regularly shifted toward the higher temperature direction with the decrease in EVM content (Figure 6), revealing that the heat storage behavior of PCE-CPCMs was significantly affected, which might have been caused by the different degrees of pore confinement and surface interactions of EVM. In the PCE-CPCMs solidification process, the confinement and surface interactions effects restricted the motion of PEG molecular chains, resulting in the inhibition of the thickening of thin lamellar PEG crystallites into stable crystal. Researchers have shown that the phase change temperature of PEG is a function of its crystallite thickness, so higher crystallite thickness would cause an increase in phase change temperature [23,32]. Hence, the PCE-CPCMs containing less EVM showed weaker inhibition effects, which led to higher crystallite thickness of the PEG, thus the phase change temperature was higher (PCE1.59 < PCE3.30 < PCE5.20 < PCE7.09). The results were consistent with the solidification process for the PCE-CPCMs melting process.

Based on our previous works [13,23], the phase change characteristics of PCE-CPCMs without CNTs (PEG-EVM) and without EVM (PEG-CNTs) are summarized in Table 4. EVM significantly affected the latent heat of PEG, as demonstrated by the experimental latent heat being significantly lower than the calculated value, perhaps due to pore confinement and surface interactions. Conversely, CNTs fillers showed little effect as the experimental and calculated latent heat were very close. In this work, the PCE-CPCMs latent heat increased with increasing CNTs weight fractions (Figure 6b and Table 3), which was mainly attributed to the decreasing inhibition effect of EVM and the increasing adsorption capacity of PEG. The pore confinement and strong surface interactions of EVM inhibited the latent heat of PCE-CPCMs because the PEG molecular chains confined in the pores of EVM or adsorbed by the surfaces of EVM were amorphous or mesomorphic phase and could not crystallize perfectly, which could hardly contribute to the latent heat [33,34]. To better understand the effects of confinement and surface interactions on the latent heat, the effective ratio ($\phi_{e}$) and inhibited ratio ($\phi_{i}$) of the latent heat of PCE-CPCMs are defined in Equations (1) and (2), respectively.

(1) $$\phi_{e}=\frac{H_{PCE-CPCMs}}{\omega H_{PEG}}\times 100\%$$

(2) $$\phi_{i}=(1-\phi_{e})\times 100\%$$

where *H_PCE-CPCMs_* and *H_PEG_* represent the latent heats of PCE-CPCMs and PEG, respectively, and *ω* is the adsorption capacity of PEG. The calculated results are shown in Table 5. The effective ratio clearly increased with decreasing EVM content, whereas the inhibited ratio was opposite, indicating that the inhibition on the latent heat of PCE-CPCMs was weakening. Notably, the inhibited ratio in the melting process was larger than that in the solidification process. The probable reason for this observation was that the EVM played another role in addition to the inhibition effect: the large specific surface area could act as a heterogeneous nucleation center, thus providing numerous nucleation sites for promoting the crystallization of PEG, which was conducive to the increased latent heat during the solidification process. In summary, the latent heat of PCE-CPCMs was enhanced via an efficient synergistic effect between EVM and CNTs.

### 3.6. PCE-CPCMs Thermal Conductivity 

**Figure 7:**
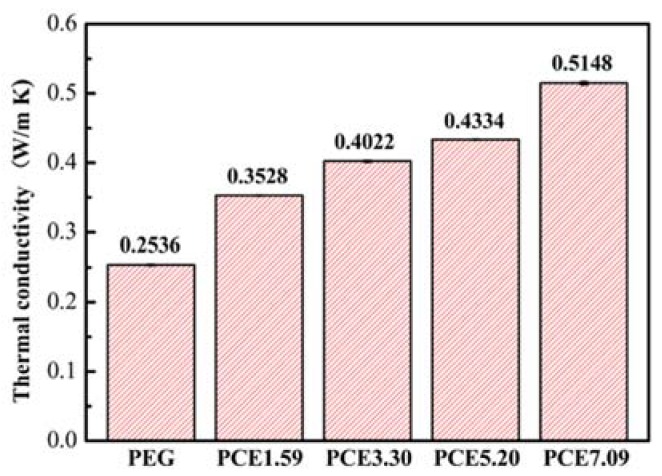
Thermal conductivity of PEG and PCE-CPCMs.

The PCE-CPCMs thermal conductivity determined the heat storage or release efficiency. Figure 7 shows the changes in the thermal conductivity of PCE-CPCMs with increasing CNTs content. The PEG showed a low thermal conductivity of 0.2536 W/(m·K), which was close to the values reported in the literature [13,35]. Compared with the thermal conductivity of PCE-CPCMs without CNTs (0.27 W/(m·K)) [23], the values for PCE1.59, PCE3.30, PCE5.20, and PCE7.09 were 0.3528, 0.4022, 0.4334, and 0.5148 W/(m·K), respectively, indicating that the CNTs fillers significantly improved the PCE-CPCMs heat transfer rate. Compared with the pure PEG, the thermal conductivity enhancement ratio of PCE1.59, PCE3.30, PCE5.20, and PCE7.09 were 39.12%, 58.60%, 70.90%, and 103.00%, respectively, which were dependent on the CNTs filler content. The close interfacial interaction between the PEG and entangled CNT network could efficiently decrease the interfacial thermal resistance, resulting in the formation of rapid thermally conductive pathways, which were responsible for the thermal conductivity enhancement of the PCE-CPCMs. Usually, the thermal conductivity of composite PCMs increases with the increase in filler content, whereas the latent heat of both the melting and solidification processes decreased monotonically [2,3,36,37,38]. In this work, the latent heat and thermal conductivity of the prepared PCE-CPCMs were simultaneously enhanced via the efficient synergistic effect between EVM and CNTs. EVM acted as the encapsulation material to prevent the leakage of melted PEG and maintained good form stability, whereas the pore confinement and surface interactions inhibited the latent heat of PEG. Moreover, the inhibition effect weakened with decreasing EVM content. The numerous surfaces of CNTs adsorbed the melted PEG, thus the adsorption capacity of PCE-CPCMs increased with increasing CNT content. Moreover, CNTs had little effect on the latent heat of PEG. More importantly, CNT fillers significantly improved the heat transfer rate of PEG.

### 3.7. PCE-CPCMs Heat Storage and Release Properties 

**Figure 8:**
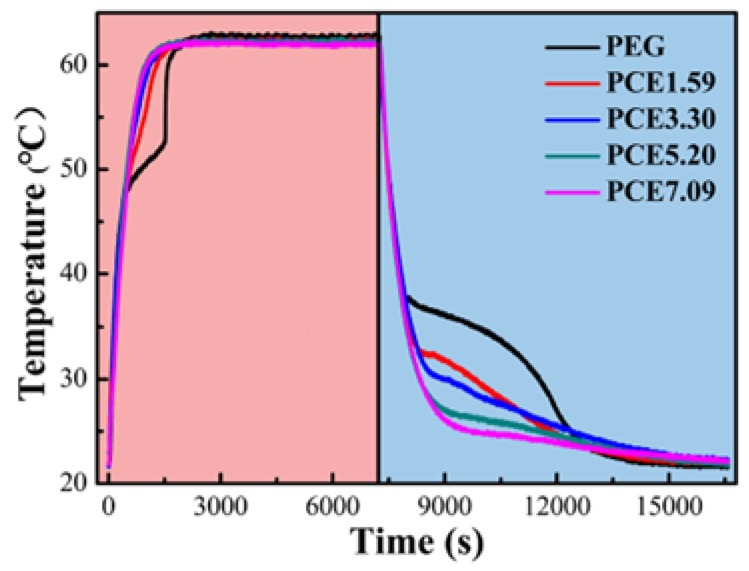
Time-temperature curves of PEG and PCE-CPCMs during the heating and cooling processes.

The heat storage and release properties of PEG and PCE-CPCMs were evaluated using their time-temperature curves (Figure 8). During the heating process, the PEG exhibited an obvious temperature platform, which was ascribed to the PEG solid–liquid phase change. However, the significant platform of PCE-CPCMs did not appear, which was mainly attributed to the pore confinement and surface interactions of EVM, whereas the heat storage process was still completed as confirmed by the DSC results in Figure 6 and Table 3. During the cooling process, obvious temperature platforms were observed for both PEG and PCE-CPCMs, indicating a large amount of stored thermal energy was released in the PEG liquid–solid phase change. By comparing the heat storage and release platform of PCE-CPCMs during the heating and cooling processes, we deduced that the inhibition effect of EVM on the heat storage and release properties of PCE-CPCMs during the melting process was stronger than that during the solidification process due to the crystallization-promoting effect, which was consistent with our analysis of the above DSC results. Moreover, compared with PEG, the PCE-CPCMs temperature both rose and fell more rapidly, indicating that the heat storage and release rate of PCE-CPCMs was considerably increased due to their improved thermal conductivity.

### 3.8. PCE-CPCMs Thermal Stability 

**Figure 9:**
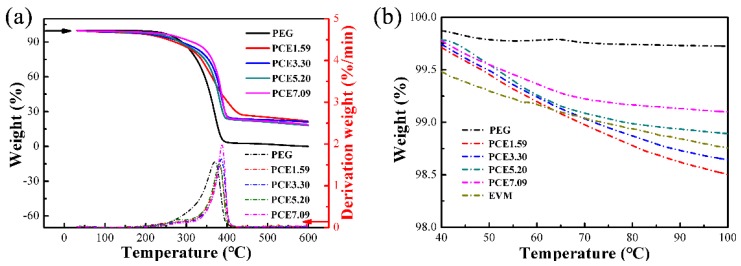
(**a**) Thermogravimetric analysis-derivative thermogravimetry (TGA-DTG) curves of PEG and PCE-CPCMs from 35 to 600 °C, and (**b**) the enlarged TGA curves from 40 to 100 °C.

The thermal stability of PCE-CPCMs was investigated using TGA and derivative thermogravimetry (DTG) (Figure 9). We observed that the PEG showed a one-step degradation process when heated to 600 °C and only a 0.07% residue was obtained. The PCE-CPCMs exhibited similar thermal stability characteristics as PEG. The weight loss percentages of PCE1.59, PCE3.30, PCE5.20, and PCE7.09 were 75.96%, 77.67%, 80.02%, and 79.16%, respectively, which was mainly attributed to the decomposition of PEG. These weight loss percentages were close to the weight fractions of PEG in Table 2. The maximum weight loss rate of PCE-CPCMs occurred at 380–388 °C, which was higher than PEG, indicating that the synergy between EVM and CNTs affected the thermal stability characteristics of PCE-CPCMs. In the designed working temperature of ca. 100 °C, as shown in Figure 9b, the weight loss percentages of PEG and EVM were 0.27% and 1.24% (resulting from the liberation of the absorption water), respectively. The weight loss percentages of PCE1.59, PCE3.30, PCE5.20, and PCE7.09 were 1.49%, 1.36%, 1.11%, and 0.90%, respectively, which was mainly attributed to the weight loss of PEG and EVM, indicating that the PCE-CPCMs exhibited desirable thermal stability.

### 3.9. PCE-CPCMs Thermal Reliability 

**Figure 10:**
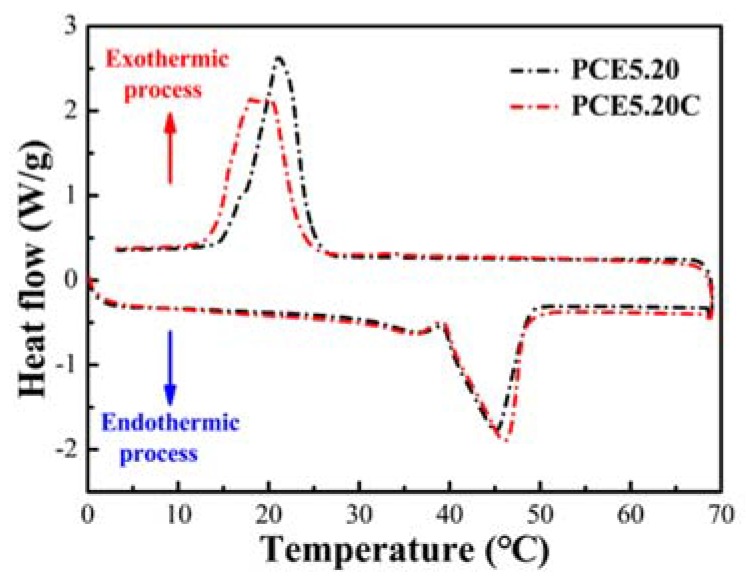
DSC curves of PCE5.20 before and after 100 thermal cycles.

**Figure 11:**
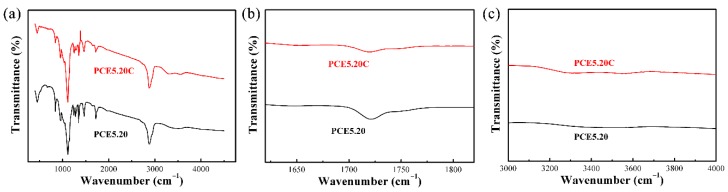
(**a**) FT-IR spectrum of PCE5.20 before and after 100 thermal cycles; (**b**) enlarged (**a**) at 1620–1820 cm^−1^; and (**c**) enlarged (**a**) at 3000–4000 cm^−1^.

The thermal reliability of fs-CPCM, after experiencing large number of thermal cycles, is an important characteristic for the long-term use of composite PCMs, and their heat storage performance and chemical structures should be stable. Figure 10 shows the DSC curves of PCE5.20 and PCE5.20C. No obvious difference was observed in the endothermic and exothermic curves of PCE5.20 and PCE5.20C except for the slight change in phase change temperature. After 100 thermal cycles, the weight loss of PCE5.20 was only 1.39%, and the latent heat changed by 3.4% during the melting process and 2.1% during the solidification process, revealing that no obvious degradation in heat storage capacity occurred. Although the oxidation of PEG (peak at 1720 cm^−1^) still existed in the PCE5.20C spectrum, the other absorption peaks, including O-H at 3421 cm^−1^, almost remained unchanged (Figure 11), indicating the good chemical compatibility of PCE5.20 and stable chemical structure after 100 thermal cycles. Based on the above results, we concluded that the fabricated PCE-CPCMs exhibited excellent thermal reliability within at least 100 thermal cycles for practical applications.

## 4. Conclusions

In this work, we reported a series of PCE-CPCMs with large adsorption capacities, and simultaneously enhanced latent heat and heat transfer. The PCE-CPCMs heat storage behavior results showed that the decreasing inhibition effect of EVM and increasing adsorption capacity of PEG were responsible for the increase in latent heat of PCE-CPCMs of 19.4–83.9 J/g during the melting process and 46.2–104.2 J/g during the solidification process. Pore confinement and surface interactions of EVM inhibited the phase change process of PCE-CPCMs, with an inhibition ratio of 85.88–41.77% during the melting process and 63.05–20.53% during the solidification process. The crystallization promoting effect strengthened the inhibition during the melting process compared to the solidification process. The PCE-CPCMs heat transfer efficiency significantly improved due to the CNTs fillers and was dependent on its content (thermal conductivity: 0.3528–0.5148 W/(m·K), enhancement ratio: 39.12–103.00%). The desirable chemical compatibility, heat storage and release properties, and thermal stability and reliability of PCE-CPCMs are beneficial to their practical applications. Based on previous research [18,19,20,21], PCE-CPCMs showed comparable properties to the reported composite PCMs, so they could be considered as a potential candidate in building-integrated solar energy conversion applications.

## Figures and Tables

**Table 1:** Chemical composition of the expanded vermiculite (EVM).

Constituent	SiO_2_	Al_2_O_3_	Fe_2_O_3_	MgO	CaO	K_2_O	H_2_O
Ratio (wt %)	43.2	12.68	4.56	24.2	0.96	5.95	7.6

**Table 2:** Components of the prepared polyethylene glycol (PEG)-carbon nanotubes (CNTs) with expanded vermiculite (EVM) form-stable composite phase change materials (PCE-CPCMs).

PCE-CPCMs	PEG (g)	CNT (g)	EVM (g)	PEG Weight Fractions (wt %)	CNT Weight Fractions (wt %)	EVM Weight Fractions (wt %)
PCE1.59	7.5360	0.1541	2.0025	77.75	1.59	20.66
PCE3.30	9.1336	0.3800	2.0027	79.31	3.30	17.39
PCE5.20	12.3063	0.7849	2.0030	81.53	5.20	13.27
PCE7.09	14.3588	1.2485	2.0022	81.54	7.09	11.37

**Table 3:** Phase change characteristics of PEG and PCE-CPCMs ^a^.

Sample	Melting Process		Solidification Process	
	T_M_ (°C)	H_M_ (J/g)	T_S_ (°C)	H_S_ (J/g)
PEG	53.27	176.7	32.28	160.8
PCE1.59	41.88	19.4	11.81	46.2
PCE3.30	42.80	25.6	16.23	58.1
PCE5.20	45.09	45.1	21.09	77.5
PCE7.09	47.98	83.9	27.26	104.2

^a^ T_M_ and H_M_ are the phase change temperature and latent heat in melting process, respectively; and T_S_ and H_S_ represent the same but for the solidification process.

**Table 4:** Phase change temperature, measured and calculated latent heat of PCE-CPCMs without CNTs (PEG-EVM) and without EVM (PEG-CNTs) during the melting and solidification processes [13,23] ^a^.

Sample (Mass Ratio)	Melting Process			Solidification Process		
	T_M_ (°C)	H_M_ (J/g)	H_C_ (J/g)	T_S_ (°C)	H_S_ (J/g)	H_C_ (J/g)
PEG-EVM (68.59:31.41)	57.11	95.15	137.46	32.62	82.48	127.66
PEG-CNTs (90:10)	55.30	165.4	165.1	43.63	151.6	150.8
PEG-CNTs (92:8)	55.76	169.4	168.7	43.25	154.6	154.2
PEG-CNTs (94:6)	56.03	172.4	172.4	43.03	157.6	157.5
PEG-CNTs (96:4)	56.32	176.4	176.1	42.81	160.6	160.9
PEG-CNTs (98:2)	56.68	179.4	179.7	42.66	163.6	164.2

^a^ H_C_ is the calculated latent heat (the product of PEG latent heat and its adsorption capacity in composite PCMs).

**Table 5:** Effective ratio ($\phi_{e}$) and inhibited ratio ($\phi_{i}$) of latent heat of PCE-CPCMs.

Sample	Melting Process		Solidification Process	
	$\phi_{e}\;(\%)$	$\phi_{i}\;(\%)$	$\phi_{e}\;(\%)$	$\phi_{i}\;(\%)$
PCE1.59	14.12	85.88	36.95	63.05
PCE3.30	18.27	81.73	45.56	54.44
PCE5.20	31.31	68.69	59.12	40.88
PCE7.09	58.23	41.77	79.47	20.53
